# Towards economic and sustainable production of poly-3-hydroxybutyrate by *Halomonas boliviensis* using as feedstock industrial residues of seaweed *Gelidium corneum*

**DOI:** 10.3389/fmicb.2026.1872452

**Published:** 2026-06-25

**Authors:** Vishwanath S. Khadye, Rodrigo Costa, M. Teresa Cesário

**Affiliations:** 1 iBB- Institute for Bioengineering and Biosciences, Department of Bioengineering, Instituto Superior Técnico, Universidade de Lisboa, Lisboa, Portugal; 2 Associate Laboratory i4HB-Institute for Health and Bioeconomy, Instituto Superior Técnico, Universidade de Lisboa, Lisboa, Portugal

**Keywords:** enzyme recycling, fed-batch fermentation, *Gelidium corneum*, *Halomonas boliviensis*, poly-3-hydroxybutyrate, seaweed hydrolysates, unsterile conditions, bioplastics

## Abstract

The production of the biopolymer poly-3-hydroxybutyrate (P3HB) by the halophile *Halomonas boliviensis* in fed-batch cultivations using bench-scale bioreactors was re-examined aiming to improve productivity and decrease production costs. To improve productivity, controlled feeding of glucose and nitrogen source, and supplementation of extra monosodium glutamate (MSG), phosphate, and trace elements were assessed. Non-aseptic conditions were investigated as a strategy to reduce costs related to medium and equipment sterilization. Further, to reduce production costs, glucose-rich hydrolysates derived from industrial residues of the red macroalga *Gelidium corneum* were used as an alternative carbon source to glucose. These hydrolysates were produced through hydrothermal pretreatment to remove residual agar followed by enzymatic hydrolysis. Enzyme recycling was evaluated as a strategy to improve process economics. After hydrolysis, the enzymatic cocktail was recovered using an ultrafiltration membrane and reused in the next batch. Supplementation of trace elements and increased phosphate addition contributed to a significant increase of cell dry weight (CDW), P3HB titer and overall P3HB volumetric productivity up to 103.1 ± 1.9 g/L, 70.9 ± 0.3 g/L, and 0.92 ± 0.04 g/L·h, respectively. Under non-aseptic conditions, P3HB titers and productivities were comparable to those achieved under axenic conditions. Algal hydrolysates proved to be an effective glucose source, yielding results similar to those obtained with commercial glucose. This study shows that H. boliviensis is a promising platform for P3HB production and can efficiently convert carbohydrate-rich residual biomass under non-sterile conditions.

## 1 Introduction

The use of traditional, non-biodegradable petroleum-based plastics has contributed to global greenhouse gas emissions, soil and water pollution (Ford et al., 2022; Abrha et al., 2022) and poisoning of the groundwater by formation of microplastics (Alaerts et al., 2018; Swetha et al., 2024). To tackle this problem, bio-based and biodegradable plastic synthesized by microbes utilizing sustainable raw materials like algal or plant-based biomass has emerged as suitable alternative to petrochemical-based plastic (Swetha et al., 2024; Mogany et al., 2024). Due to their high recyclability, compostability, and minimal carbon footprint, bioplastics are beneficial and environmentally friendly (Mogany et al., 2024). However, for these bioplastics to be commercially viable, they must exhibit comparable material properties and processability while remaining economically feasible (Jo et al., 2024).

The polyesters polyhydroxyalkanoates (PHAs), due to their side chains diversity, excellent biodegradability, and biocompatibility, are considered as promising alternatives to non-degradable petroleum-derived plastics (Swetha et al., 2024; Zinn et al., 2001). Among the different types of PHAs, Poly-3-hydroxybutyrate (P3HB) a homopolymer of 3-hydroxybutyric acid shows satisfactory thermal and mechanical properties (Alves et al., 2017). P3HB is naturally synthesized by diverse microbial species and it can be readily degraded in natural environmental conditions making it a promising candidate for various applications in packaging, agriculture and pharmaceutical industries (Peña et al., 2014) as an alternative to petroleum-derived plastic. P3HB synthesis by different microbial species such as: *Bacillus megaterium* (Gouda et al., 2001), *Cupriavidus necator* (Dalsasso et al., 2019; Saratale and Oh, 2015), *Zobellella denitrificans* (Asiri et al., 2020), *Azotobacter chroococcum* (Nagajothi and Murugesan, 2023), and several species of the genus *Halomonas* (Leandro et al., 2023; Quillaguaman et al., 2004; Kucera et al., 2018), has been reported.

*Halomonas* species such as *H. boliviensis* (Quillaguaman et al., 2004), *H. halophila* (Kucera et al., 2018), *H. elongata* (Leandro et al., 2023), *H. bluephagenesis* (Zhang et al., 2024), and *H. campaniensis* (Deantas-Jahn et al., 2024) have been reported to accumulate P3HB (Biswas et al., 2023; Tennakoon et al., 2023). These moderate halophiles can tolerate high salt concentrations of 3–15% (w/v) (Biswas et al., 2023, Tennakoon et al., 2023) showing optimal growth at mesophilic temperatures (25–37 °C) and wide pH range (6.0–9.0) (Trivedi and Soni, 2025; Edbeib et al., 2016; Kumar et al., 2012). These organisms use either salt-in or compatible solute strategies to maintain a constant intercellular osmotic balance under the conditions of high salt-concentration (Edbeib et al., 2016). Researchers have started exploring *H. boliviensis* for the production of P3HB due its ability to accumulate high concentrations of biopolymer (50–88% of the dry cell weight) (Quillaguaman et al., 2004). Also, *H. boliviensis* is known to metabolize different monosaccharides, disaccharides, and polysaccharides as a carbon source (Biswas et al., 2023; Edbeib et al., 2016) allowing its cultivation on various low-cost media components. Different strategies like phosphate, nitrogen or oxygen limitation in presence of excess carbon source have been explored to enhance the synthesis of P3HB by *H. boliviensis* (Bondar et al., 2022; García-Torreiro et al., 2016). Using these strategies, researchers have achieved P3HB productivities of up to 1.1-1.2 g/(L·h) while using commercial glucose as a carbon source (García-Torreiro et al., 2016; Quillaguamán et al., 2008).

Carbon source and sterilization costs account for the major limitation in industrial scale P3HB production (Akkoyunlu et al., 2024; McAdam et al., 2020). Halophiles enable the bioprocessing operations in unsterile conditions due to their tolerance to high concentrations of NaCl avoiding the problem of contaminations (Asiri et al., 2020; Wang et al., 2025). Growing halophiles on cheaper carbon-rich feedstocks such as algae or plant-derived hydrolysates (Torrejon et al., 2025; Van-Thuoc et al., 2008), domestic wastes (Trivedi and Soni, 2025; Wang et al., 2025), and industrial wastes (Bondar et al., 2022) have been explored for sustainable P3HB production. However, bioconversion of such cellulosic biomass requires hydrothermal or mild acid pretreatment to make cellulosic structures more susceptible to enzymatic attack followed by enzymatic hydrolysis to release fermentable sugars (Qi et al., 2012). Enzyme cost also becomes the major contribution to the overall cost of biomass hydrolysis-based processes and it can be effectively reduced by enzyme recycle (Xue et al., 2012). Recently, a red seaweed *Gelidium corneum* residue from the agar extraction industry was exploited as a potential carbon source (Bondar et al., 2022; Tůma et al., 2020). These residues not only contain about 30–40% (w/w) carbohydrate mainly in the form of cellulose (22–37%) which upon enzymatic hydrolysis release glucose, but also leftover agar of circa 7–12% (w/w) (Tůma et al., 2020; Mateus et al., 2024). *H. boliviensis* was cultivated in shake flasks on *G. corneum* hydrolysates generated by mild acid pretreatment followed by enzymatic hydrolysis and maximum P3HB concentration and content of 3.0 g/L and 32% (w/w), respectively (Tůma et al., 2020) were achieved. In another study, hydrolysates of *G. corneum* residues were produced applying hydrothermal pretreatment followed by enzymatic hydrolysis (Bondar et al., 2022) and used in *H. boliviensis* fed-batch cultivations in a 2-L scale bioreactor achieving P3HB concentration, content and overall volumetric productivities of 21.5 g/L and 41% (w/w) and 0.46 g/(L·h) respectively.

P3HB production by *H. boliviensis* on commercial glucose was explored earlier by our group using N- and P-limitation strategies. The effect of oxygen concentration was also studied (Bondar et al., 2022). Results suggested P-limitation along with the 5% oxygen saturation (DO %) as the best strategy. The maximum CDW, P3HB concentration, P3HB content and P3HB productivity achieved were 49.5 ± 4.9 g/L, 30.4 ± 0.7 g/L, 62.2 ± 7.3%, and 0.45 ± 0.03 g/(L·h), respectively.

The objective of this work was to first optimize media and bioprocess conditions to enhance *H. boliviensis* growth and biopolymer production. In this regard, parameters like controlled feeding of glucose and nitrogen source, monosodium glutamate (MSG), phosphate, and trace element supplementation were extensively investigated. The optimized parameters were further replicated with glucose derived from *G. corneum* residues after hydrothermal pretreatment and enzymatic hydrolysis. To reduce cost related with the manufacture of hydrolysates, the possibility to recycle the enzymatic cocktail was addressed. In addition to further decrease costs, P3HB productivity was assessed in cultivations carried out in unsterile conditions.

## 2 Materials and methods

### 2.1 Raw material and chemicals

The raw material was provided by Iberagar–Sociedade Luso-Espanhola de Colóides Marinhos S.A. (Portugal) and it consisted of the solid residue from the red macroalga *Gelidium corneum* after agar extraction. The biomass received was first washed several times with tap water to remove sand particles and other debris. The biomass was then dried at 50 °C for 24 h and stored in a closed container at room temperature, until further use. The dried biomass was subsequently grinded using variable speed rotor mill PULVERISETTE 14 classic line (FRITSCH) using 0.2 mm sieve ring to attain fine powder of biomass and was stored in closed container till use.

Commercial glucose (dextrose monohydrate), Dextropan 100 supplied by Copam-Portugal, was used as the substrate in all bioreactor cultivations. D (+) glucose anhydrous 99.5% (Fisher Chemicals) and gluconic acid sodium salt 99% (Sigma) were used as HPLC standards. For enzymatic hydrolysis of pretreated *Gelidium* biomass, Cellic Ctec3 HS was used (Novozymes). The FPU activity (129.02 ± 2.98 FPU/mL) and beta-glucosidase activity (2,130 ± 26.5 pNPU/mL) measurements were performed according to a standard protocol (Ghose, 1987).

### 2.2 Bacterial strain and storage

*Halomonas boliviensis* DSM 15516, a halophile capable of producing P3HB, was used in all cultivations. Stock cultures of *H. boliviensis* were prepared by growing cells in seed medium at 30 °C and 200 rpm for 22–24 h. Culture was stored at −80 °C in 2 mL cryovials containing 1.5 mL of grown culture (absorbance of 4.5–5.0 at 600 nm) and 0.3 mL of heat-sterilized glycerol (86–99%, Sigma).

### 2.3 Seed medium and inoculum preparation

The seed medium composition is described in Table 1. The medium components except NaCl, MgSO_4_·7H_2_O, FeSO_4_·7H_2_O and glucose were dissolved in distilled water, the pH was adjusted to 7.5 with HCl (5 M) and the solution was sterilized at 121 °C for 20 min. The stock solutions of NaCl (300 g/L), MgSO_4_·7H_2_O (100 g/L) and glucose (500 g/L) were prepared and heat-sterilized separately. The stock solution of FeSO_4_·7H_2_O (50 g/L) was filter-sterilized using 0.20 μm syringe filters. These components were then aseptically added to the medium to attain the desired concentrations. For the bioreactor inoculum preparation, the contents of four cryovials were added to 500 mL Erlenmeyer flasks containing 130 mL seed medium. The flask was incubated at 30 °C in an incubator with constant shaking at 200 rpm for 22–24 h to attain the late exponential phase. The absorbance achieved at 600 nm was approximately 5.0 at the moment of bioreactor inoculation.

**Table 1:** Composition of batch medium (BM) and feeds for fed-batch cultivations.

Medium components	Seed(g/L)	BM1(g/L)	BM2(g/L)	BM3(g/L)	BM4(g/L)	Feed 1 (g/L)	Feed 2* (g/L)
NaCl	45.0	45.0	45.0	45.0	45.0	45.0	NA
MgSO_4_·7H_2_O	2.5	5.0	5.0	5.0	5.0	5.0	NA
K_2_HPO_4_	0.55	1.5	1.5	1.5	3.0	NA	NA
NH_4_Cl	2.3	6.0	2.4	2.4	2.4	NA	47.0
MSG	3.0	20.0	20.0	20.0	20.0	NA	520.0
FeSO_4_·7H_2_O	0.005	0.005	0.005	0.005	0.005	0.125	NA
Glucose	20.0	20.0	20.0	20.0	20.0	600.0	NA
Trace elements solution	**	NA	NA	3 mL/L	3 mL/L	**	NA

NA, media component not added; *Final volume of 100 mL; **Component added based on the strategy used and mentioned in the text wherever necessary.

The fermentation medium composition for P3HB production in 2-L bioreactor is given in Table 1. A concentrated K_2_HPO_4_ solution was sterilized in the bioreactor while concentrated solutions of MSG, NH_4_Cl, MgSO_4_·7H_2_O (100 g/L), NaCl (300 g/L) and glucose (500 g/L) were autoclaved separately and added to the bioreactor aseptically to attain the desired concentrations. The concentrations of MSG and K_2_HPO_4_ components were varied (Table 1) based on the strategy used. Antifoam was connected to the bioreactor to control foaming. The feed medium for fed-batch phase was prepared separately and its composition is described in Table 1. The feed solution was connected after initial batch cultivation from 9–24 h, depending upon the cultivation strategy, with feeding rate controlled to prevent excess glucose in the fermenter. The trace elements solution (TES) was added to the inoculum, fermentation medium and fed-batch feed (Table 1), based on the strategy used to promote the growth of *H. boliviensis* and P3HB production. Its composition is described in Table 2.

**Table 2:** Trace elements solution (TES) composition.

Composition	Concentration (g/L)
FeCl_3_·6H_2_O	9.7
CaCl_2_·2H_2_O	7.8
CuSO_4_·5H_2_O	0.156
CoCl_2_·6H_2_O	0.119
NiCl_2_·6H_2_O	0.118
HCl	100 mM

### 2.4 Bioreactor cultivations

Fed-batch studies of *H. boliviensis* were performed in 2 L glass bench-top bioreactor (New Brunswick BioFlow/CelliGen® 115) with 1.3 L initial working volume and 10% inoculum volume. The culture pH was maintained at 7.5 ± 0.10 with 30% (v/v) NH_4_OH and 2 M H_2_SO_4_. The temperature was maintained at 30 °C with heating jacket and cold water circulating through the cool loop. The dissolved oxygen (DO) was maintained at 5% saturation with the help of agitation cascade. The aeration was maintained at 1.0 v.v.m. by passing compressed air through the sterile filter (0.2 μM pore size), and the agitation speed was set in cascade with the DO, starting at 200 RPM and setting maximum at 1200 RPM which is the maximum speed allowed by the system. Dissolved oxygen and pH values were determined on-line using the DO probe and pH probe (METTLER TOLEDO). The process was operated by BioCommand Batch Control software with enabled monitoring, control, and data acquisition. During fed-batch, feed 1 (Table 1) was added to the fermenter with feeding rate varied to maintain glucose concentration above 25–30 g/L. In the case of feed 2, its addition was initiated immediately after inoculation until 9 h EFT with feeding rate controlled to add approximately 6 g of MSG per hour to the fermenter.

When *G. corneum* hydrolysates were used as feed, a bioreactor Eppendorf Bioflo 120 of 5 L working volume was used. The same set-points were used as above except for the initial batch volume of 2 L. BM4 medium was used in the batch phase and feed 2 was added till 9 h cultivation after which, instead of feed 1, the hydrolysate supplemented with NaCl, MgSO_4_·7H_2_O and FeSO_4_·7H_2_O was added.

All bioreactor cultivations were performed in duplicates.

### 2.5 Analytical methods

Culture samples were collected at different time points throughout the time of *H. boliviensis* cultivation. Samples were analyzed for OD_600nm_, cell dry weight (CDW), P3HB concentration, and the presence of residual glucose and organic acids formed.

#### 2.5.1 Cell dry weight

The CDW was determined by centrifuging 1.2 mL culture sample at 10000 rpm for 5 min in previously dried and weighed micro-centrifuge tubes. The supernatant was discarded and the cell pellet was washed three times with distilled water. After final round of centrifugation, the cell pellet was dried at 60 °C in an oven until the constant weight is achieved.

#### 2.5.2 Sugar and organic acid quantification

A high-performance liquid chromatography (HPLC) Hitachi LaChrom Elite system equipped with a Rezex ROA-Organic acid H + 8% (300 mm × 7.8 mm) column, a Hitachi L-2490 refraction index (RI) detector for sugars, and a Hitachi L-2420 UV-VIS detector for organic acids, was used for offline determination of residual glucose and gluconic acid formed during the entire fermentation process. The column temperature was maintained at 65 °C with the help of externally connected column heater (Croco-CIL 100-040-220P; 40 cm × 8 cm × 8 cm; 30–99 °C). The sample volume was 20 μL. A 5 mM H_2_SO_4_ solution was used as a mobile phase at a flow rate of 0.5 mL/min. Quantification of glucose and gluconic acid were done based on the calibration curves obtained under the same conditions.

#### 2.5.3 Quantification of poly-3-hydroxybutyrate

For quantification of P3HB, depending upon the cell growth and P3HB produced, an adequate volume (1.2, 0.6, 0.3, 0.15 or 0.075 mL) of culture broth was withdrawn at different sampling points to a microcentrifuge tube and centrifuged. The cell pellet was washed 3-fold with distilled water followed by centrifugation at 5000g for 5 min stored at −20 °C until analysis. The cell pellet was then subjected to acidic methanolysis to convert the polymer into stable and volatile hydroxycarboxylic acid methyl esters, to be further detected by gas chromatography. The methanolysis protocol was followed as described by Bondar et al. (2022) and P3HB concentration was determined by gas chromatography (Hewlett Packard 5,890 series II) using an FID detector. The HP-Ultra 2 column (Agilent J&W Scientific) of 25 m length and 0.2 mm internal diameter was used. The oven, injector and detector temperatures were maintained at 100 °C, 150 °C, and 200 °C, respectively. Calibration curves were obtained using samples of P3HB produced previously (purity 98.2%), which were subjected to the same methylation process as the cells. The hexanoic acid (99% purity, Thermo Scientific) was used as an internal standard.

### 2.6 Preparation of the *Gelidium corneum* hydrolysate

#### 2.6.1 Hydrothermal pretreatment

The finely grinded biomass from *Gelidium* residue (300 g dry weight basis) was mixed with 3 L distilled water (10% solid loading) in a 5.0 L Stirred Pressurized Bench Top Reactor (Amar equipment Pvt. Ltd., Mumbai, India) and it was subjected to a hydrothermal pretreatment to remove residual agar. The pretreatment reaction was performed at 170 °C for 20 min under constant stirring at 300 rpm and 7 bar pressure. At the end of the pretreatment, the resulting slurry was filtered (at temperatures above 80 °C) to separate the biomass from the liquid and the biomass was dried overnight at 55 °C. The dried residues were then subjected to enzymatic hydrolysis.

#### 2.6.2 Enzymatic saccharification of pretreated biomass and enzyme recycling

The enzymatic hydrolysis of pretreated *Gelidium* biomass was performed at 10% solids loading (w/v) and enzyme (Cellic Ctec3 HS) dosage of 130 FPU/g_biomass_ in 1 L acidified water (pH adjusted to 5.0 ± 0.2). The enzymatic hydrolysis was carried out by incubating the flask at 50 °C and constant shaking at 150 rpm, for 4 h. The concentration of the reducing sugar released at the end of the hydrolysis was analyzed by HPLC. The slurry was centrifuged at 8000 rpm for 20 min to separate the biomass from the supernatant containing the glucose. The supernatant was further lyophilized (Alpha 1–4 LSCbasic, CHRIST) to obtain the *Gelidium* hydrolysate in powder form. This hydrolysate powder was stored in airtight container at −20 °C until further use.

In the enzyme recycling assay, after the enzymatic hydrolysis and separation of solid–liquid, the supernatant was first filtered through a cotton cloth to remove biomass debris. The filtrate containing a mixture of glucose and enzyme was then passed through a hollow fiber ultrafiltration membrane of 1,600 cm^2^ area with 3 KDa MWCO (Repligen) for separation of soluble sugars (in permeate fraction) and soluble enzymes (in retentate fraction). The enzyme activity profile (FPU and beta-glucosidase activity) was measured (Ghose, 1987) to calculate the enzyme recovery using Equation 1.


$$\text{Recovery of activity}(\%)=(\frac{\text{Measured activity}}{\text{Initial activity}})\times 100$$ (1)

The ultrafiltration retentate was recycled for fresh biomass hydrolysis (10% loading) along with the addition of fresh enzyme (Cellic Ctec3 HS), based on the enzyme recovery in the earlier hydrolysis cycle, to achieve standard enzyme loading conditions, i.e., 130 FPU/g of biomass. This enzyme recycle process was repeated three times.

### 2.7 Production of P3HB by *Halomonas boliviensis* under non-sterile conditions

P3HB was produced under unsterile conditions in a 2-L bioreactor containing 1.17 L of BM4 medium (Table 1). No medium components, tubing and bioreactor underwent sterilization. The seed culture (130 mL) of *H. boliviensis* was aseptically grown and added at 10% (v/v). The aeration was provided at a rate of 1.0 v.v.m. and the dissolved oxygen was maintained at 5% saturation by agitation cascade. The medium pH was maintained at 7.50 ± 0.10 by adding 30% (v/v) NH_4_OH and 2 M H_2_SO_4_. The fermentation was carried out for 144 h effective fermentation time (EFT) at 30 °C. The CDW of *H. boliviensis* and P3HB concentration was measured as described in Section 2.5.

## 3 Results

### 3.1 Fed-batch cultivation of *Halomonas boliviensis* for P3HB production

**Figure 1:**
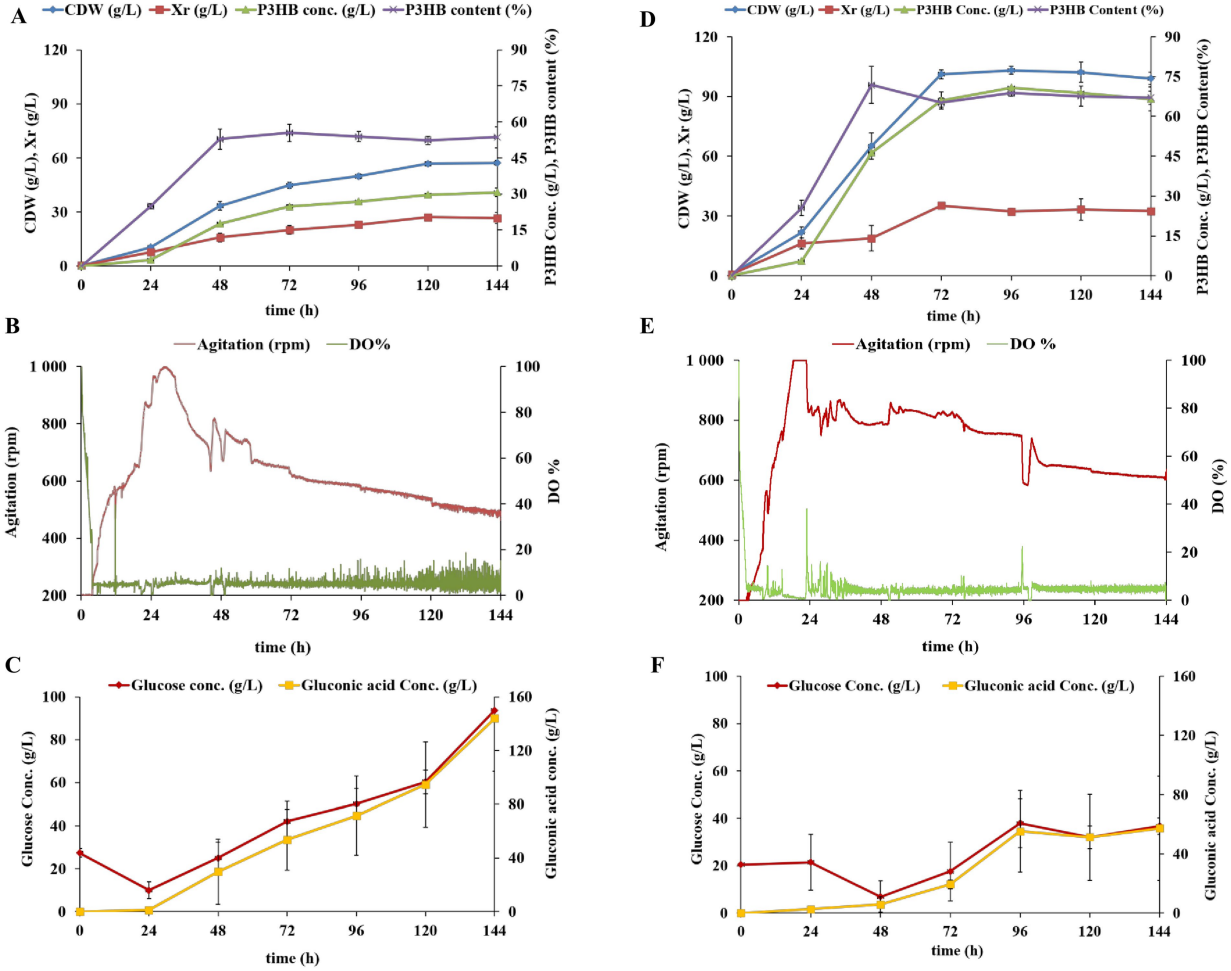
*H. boliviensis* fed-batch cultivations in 2.0 L bioreactor under controlled and optimized conditions. Panels **(A-C)** represent the results of the cultivation with BM1 batch medium and Feed 1 while Panels **(D-F)** show the results of the cultivation with BM4 medium and Feeds 1 and 2. Panels **(A,D)** depict *H. boliviensis* growth and production profile based on Cell dry weight (CDW), residual cell concentration (Xr), P3HB concentration and P3HB content; panels **(B,E)** show the automatic acquired agitation and dissolved oxygen profile during cultivation and panels **(C,F)** depict the residual glucose concentration and formed gluconic acid during cultivation.

Growth and P3HB production by *H. boliviensis* DSM 15516 were carried out on BM1 batch media along with feed 1 used for fed-batch cultivation (Table 1). Phosphate limitation strategy (Bondar et al., 2022) was followed for P3HB production. Fed-batch mode was started at 24 h EFT with addition of feed 1 (Table 1) and the feed rate was adjusted to maintain the glucose concentration above 25–30 g/L to maintain excess carbon source in the medium for P3HB production under phosphate-limitation. The DO% saturation was maintained at 5% throughout the fermentation run with the help of agitation cascade. This run is further referenced as the control run. The growth and P3HB production profile of *H. boliviensis* in BM1 media is represented in Figures 1A,B. The maximum CDW of 59.8 ± 1.0 g/L and Xr (residual cell mass) of 26.6 ± 3.1 g/L were achieved after 144 h of cultivation. Because P3HB is intracellular, to be able to assess cell concentration and cell growth, residual cell biomass (Xr) was calculated as the difference between the CDW and P3HB concentrations. P3HB concentration and contents reached up to 30.6 ± 1.8 g/L and 51.3%, respectively, with P3HB volumetric productivity of 0.21 ± 0.013 g/(L·h). Maximum productivity was nevertheless attained at 72 h cultivation with a value of 0.34 g/(L·h). A summary of these values is given in Table 3. This table reports maximum concentrations attained at the end of the cultivation (144 h) and values calculated at 72 h cultivation where maximum overall P3HB productivities were observed. Production of gluconic acid was observed during these cultivations and it increased after 48 h cultivation reaching up to 144.0 ± 1.2 g/L (Figure 1C). Gluconic acid production by *H. boliviensis* has been observed before by Bondar et al., (2022) and it connected to glucose concentration in the medium. The exact reason why cells excrete this sugar acid to the medium is not known, however a putative reason might be related with an overflow metabolism caused by a limited ability to catabolize glucose (Pastor et al., 2013).

**Table 3:** Overall comparison between control and optimized fermentation conditions to achieve maximum growth and PHB production.

Fermentation condition	Values at 72 h EFT					Values at 144 h EFT				
	CDW (g/L)	Xr (g/L)	PHB conc. (g/L)	PHB content (%)	PHB productivity (g/L·h)	CDW (g/L)	Xr (g/L)	PHB conc. (g/L)	PHB content (%)	PHB productivity (g/L·h)
Control run	44.8 ± 1.8	20.0 ± 2.5	24.8 ± 0.6	55.4 ± 3.7	0.34 ± 0.01	57.2 ± 1.3	26.6 ± 3.1	30.6 ± 1.8	53.7 ± 4.4	0.21 ± 0.01
N-source	45.7 ± 3.0	17.1 ± 2.2	28.6 ± 5.2	62.1 ± 7.3	0.4 ± 0.07	58.6 ± 2.1	18.9 ± 1.9	39.7 ± 0.2	67.8 ± 2.0	0.28 ± 0.01
TES	61.1 ± 4.2	23.5 ± 1.8	37.6 ± 2.4	61.6 ± 0.3	0.52 ± 0.03	71.9 ± 2.2	26.1 ± 2.5	45.8 ± 0.4	63.7 ± 2.4	0.32 ± 0.003
Optimized run	101.2 ± 2.3	35.2 ± 0.9	65.9 ± 3.2	65.15 ± 1.7	0.92 ± 0.04	99.0 ± 3.1	32.5 ± 1.4	66.5 ± 4.5	67.1 ± 2.4	0.46 ± 0.03

### 3.2 The impact of media components and feeding strategy optimization on growth and P3HB production by *Halomonas boliviensis*

The cell growth was observed to be slower in BM1 media taking approximately 120 h EFT to achieve maximum Xr (Figure 1A). This results in an overall reduction of the volumetric productivity of P3HB. The P3HB concentration attained was comparable to that achieved in the previous study using this strain (Bondar et al., 2022). Hence, to enhance *H. boliviensis* growth and P3HB volumetric productivity, changes in media components and feeding strategy were assessed as described below.

#### 3.2.1 Nitrogen source

To assess if *H. boliviensis* growth could be further improved, the fed-batch cultivation was carried out with modifications in concentrations and feeding strategy of the nitrogen source. Following the approach of Quillaguamán et al. (2008), lower initial concentrations of NH_4_Cl with subsequent intermittent feeding of this nutrient was carried out. Also, a 3-fold increase of the total amount of added MSG was performed. To attain this, BM2 medium with a lower NH_4_Cl concentration (40% of the initial concentration) was used in the batch phase. The remaining amount of NH_4_Cl was added using feed 2 (Table 1). Feed 2 also included additional MSG, with its supplementation beginning immediately after inoculation at an approximate rate of 6.0 g/h, being completed by 9 h EFT. After 9 h, addition of feed 1 was started with a feeding rate adjusted to maintain glucose concentration above 25–30 g/L throughout the cultivation. The CDW and Xr after 144 h EFT reached up to 58.6 ± 2.1 g/L and 18.9 ± 1.9 g/L, respectively (Supplementary Figure S1A) and did not show any significant improvement in comparison to control conditions (Figure 1). However, P3HB concentration and P3HB content reached up to 39.7 ± 0.2 g/L and 67.8 ± 2.0%, respectively (Supplementary Figure S1A) which represents 29.7% increase in P3HB concentration and absolute increment of 14.1% in P3HB content, compared to control run (Figure 1A). The overall volumetric P3HB productivity at 144 h EFT was 0.28 ± 0.01 g/(L·h) but maximum overall productivity was attained at 72 h cultivation with a value of 0.4 ± 0.07 g/(L·h) (Table 3).

#### 3.2.2 Effect of trace elements solution (TES)

Addition of trace elements to the initial cultivation medium and its influence on the growth and P3HB production by *H. boliviensis* was assessed. The composition of the TES was selected based on medium composition of *H. elongata* with modifications as described in Table 2. Therefore, cultivation on BM3 (Table 1) medium (BM2 supplemented with 3 mL/L of TES) was carried out along with feed 2 addition as described in section 3.2.1. After 144 h of cultivation, maximum CDW of 71.9 ± 2.2 g/L and Xr of 26.1 ± 2.5 g/L were attained, while the maximum P3HB concentration, content and volumetric productivity of 45.8 ± 0.4 g/L, 63.7 ± 2.4% and 0.32 ± 0.003 g/(L·h) respectively were achieved (Supplementary Figure S2A). A higher maximum P3HB overall volumetric productivity of 0.52 ± 0.03 g/(L·h) attained at 72 h was also observed (Table 3).

#### 3.2.3 Effect of phosphate concentration

Phosphorus plays a vital role in cellular growth and its overall energy metabolism ultimately regulates transition from growth to biopolymer accumulation phase upon its limitation (Castagnoli et al., 2025). In the previous trials, phosphate concentration was unchanged (Table 1). Although some improvement in Xr and P3HB concentrations was observed, achieving these values required a longer fermentation time. This significantly reduced the P3HB volumetric productivity. To determine whether phosphorus availability had an effect on productivity, the phosphate concentration in the batch medium was increased to 3.0 g/L. This increase was tested in combination with supplementation of TES, MSG and slow addition of NH_4_Cl (BM4 medium, Table 1). The overall growth, P3HB production profile, glucose consumption and gluconic acid formation is represented in Figures 1D−F. *H. boliviensis* growth and P3HB production was observed to be enhanced significantly with maximum CDW, Xr, P3HB concentration, and P3HB content of 101.2 ± 2.3 g/L, 33.3 ± 0.9 g/L, 65.9 ± 3.2 g/L and 65.2 ± 1.7% respectively, attained at 72 h (Figure 1D). At this cultivation time maximum P3HB volumetric productivity of 0.92 ± 0.045 g/(L·h) was attained (Table 3).

### 3.3 Feasibility of P3HB production by *Halomonas boliviensis* under non-aseptic conditions

**Figure 2:**
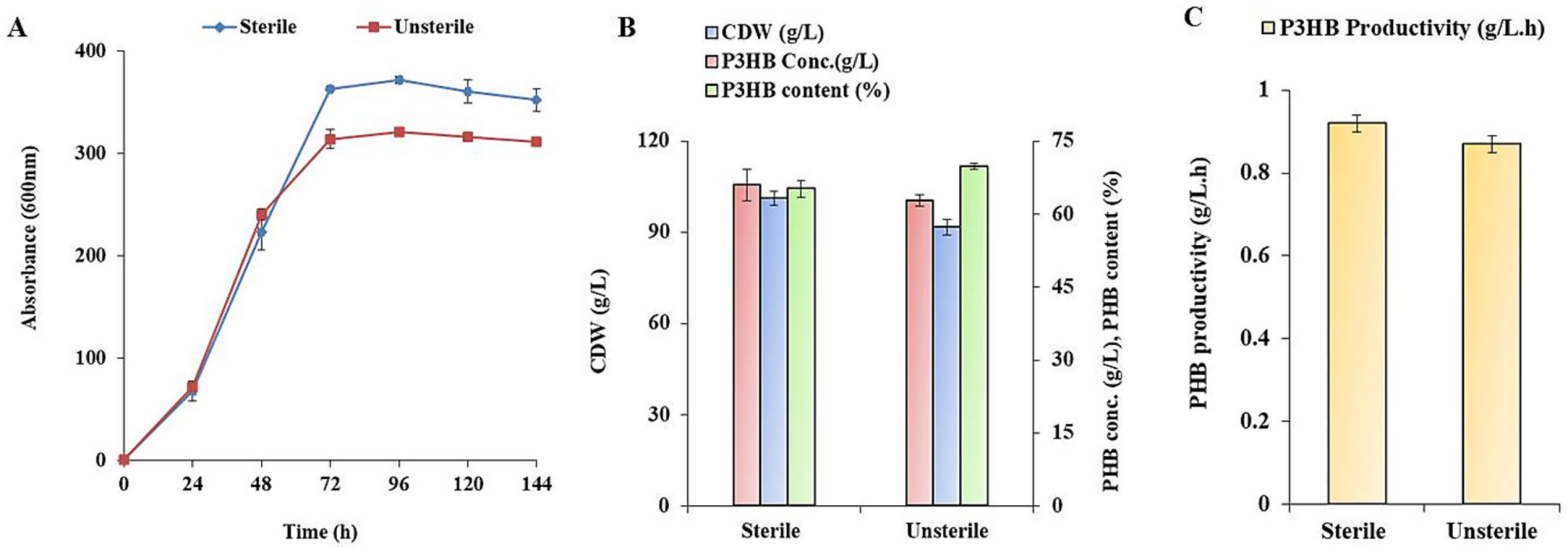
Growth and P3HB production profiles of *H. boliviensis* cultivation under sterile and unsterile conditions. **(A)** *H. boliviensis* growth in BM4 medium under sterile and unsterile conditions. **(B)** Comparison of CDW (g/L), P3HB Concentration (g/L) and P3HB contents at 72 h EFT under sterile and unsterile conditions of growth. **(C)** P3HB volumetric productivity (g/L·h) comparison for sterile and unsterile conditions. The graphs show means of duplicate experiments. The error bars show the standard deviation.

The feasibility of using non-aseptic conditions for *H. boliviensis* growth and P3HB production was assessed. *H. boliviensis* was cultivated in BM4 medium under non-sterile conditions. Cultivation took place as described in section 2.7 and growth was monitored until 144 h EFT. Maximum cell growth was attained at 72 h with CDW of 90 ± 2.5 g/L and Xr of 27.2 ± 1.2 g/L and the maximum P3HB concentration and content of 62.8 ± 1.1 g/L and 69.8 ± 0.7%, respectively. The P3HB volumetric productivity was maximum at 72 h EFT and reached up to 0.87 ± 0.02 g/(L·h). These results are comparable to values attained under sterile conditions (Section 3.2.3). The overall growth and P3HB production profiles after 72 h of *H. boliviensis* cultivation under sterile and unsterile conditions are compared in Figure 2.

### 3.4 Recycling of the cellulase cocktail after enzymatic hydrolysis

**Figure 3:**
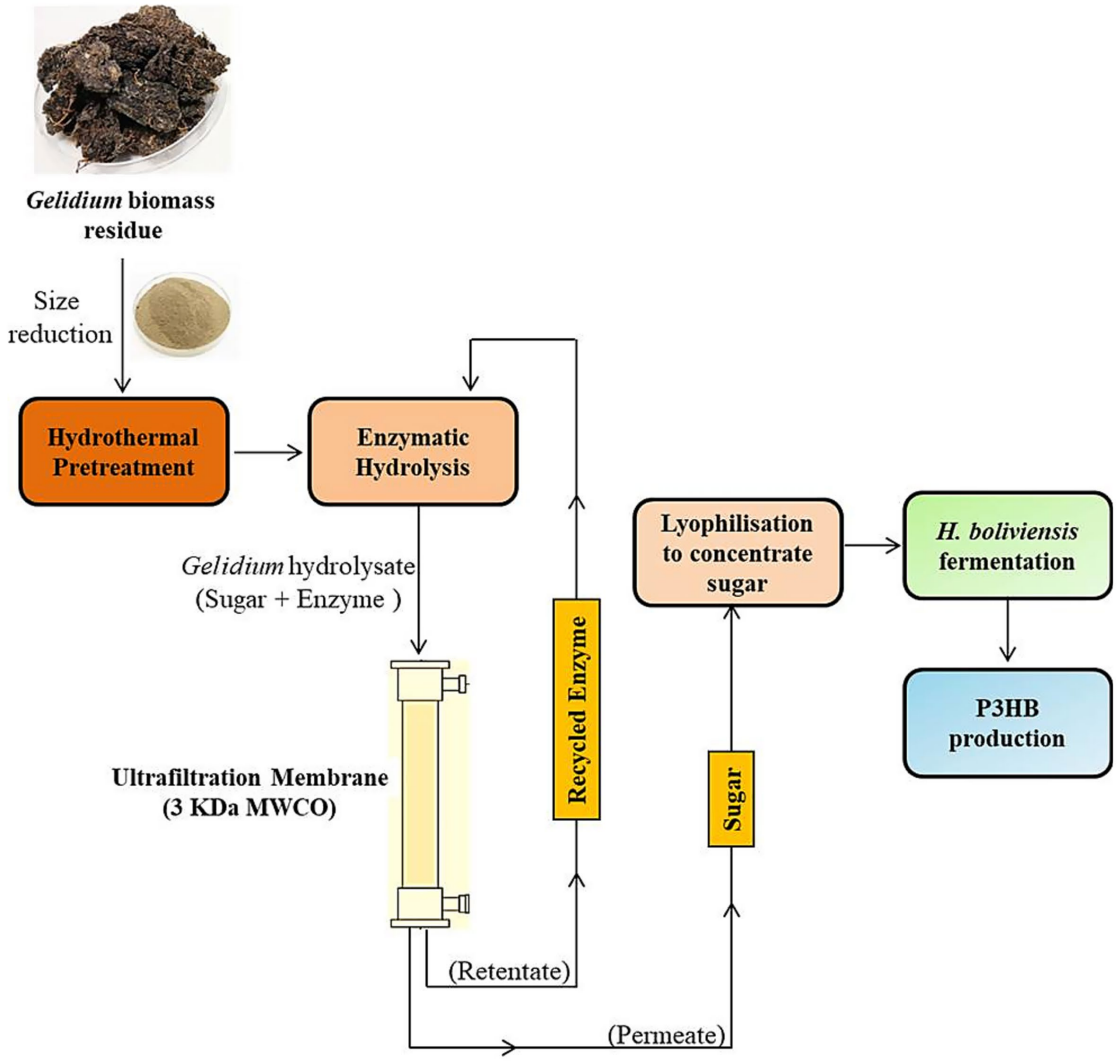
Scheme for glucose separation and enzyme recovery by ultrafiltration system for enzyme recycling.

**Figure 4:**
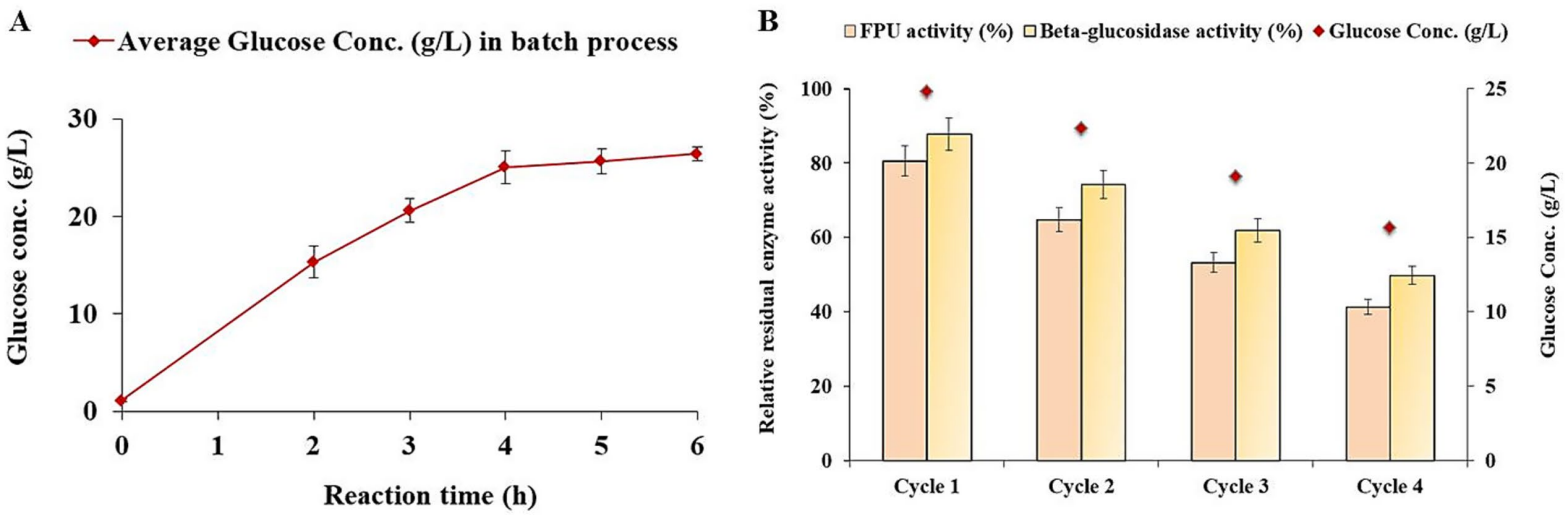
Glucose concentration and enzyme recovery during enzyme recycling process. **(A)** Glucose released during the enzymatic hydrolysis of pretreated *Gelidium* biomass using 100 g/L biomass slurry and 130 FPU/g of biomass loading. **(B)** FPU and beta-glucosidase activity recovered during each cycle of enzymatic hydrolysis and glucose titer achieved after each hydrolysis cycle.

After hydrothermal pretreatment of *Gelidium* residue biomass (section 2.6.1), the enzymatic hydrolysis took place. A high enzyme loading was used to achieve maximum hydrolysis in a shorter incubation time and so avoid sugar consumption due to microbial contamination. To recover the enzyme after hydrolysis, the liquid fraction of *Gelidium* hydrolysate was collected and filtered while the solid residue was discarded. The scheme for enzyme recycling strategy is shown in Figure 3. In the first cycle with 10% biomass loading and 130 FPU/g of enzyme loading, maximum 25.05 ± 1.65 g/L glucose concentration was achieved in 4 h of hydrolysis (Figure 4A). Only 850 mL of filtrate with glucose and enzyme mixture was recovered, which was 85% of the initial liquid volume of the hydrolysis reaction mixture. The liquid fraction was collected (section 2.6.2) and enzyme recovery was carried out by ultrafiltration. The glucose fraction was recuperated and the concentration monitored, whereas the enzyme fraction was recycled for the next round of hydrolysis. The operational stability of enzymes is a critical parameter in enzyme recycling strategy and it was monitored by measuring the enzyme activity before each utilization during enzyme recycle. The FPU and beta-glucosidase activity recovered from cycle 1 was found to be 80.6 and 87.7%, respectively, (Figure 4B) while a glucose concentration of 24.3 g/L was measured in the permeate representing 97.1% recovery of the average glucose concentration obtained after the first batch hydrolysis (Figure 4A). The enzyme recovered in the first cycle was used for the next cycle of hydrolysis of fresh biomass. It was observed that enzyme activity steadily declines after each cycle of hydrolysis and enzyme recycling (Figure 4B). The glucose concentration was also observed to be reduced (Figure 4B) with every successive cycle of hydrolysis. After 4 cycles, only 41.3% of FPU activity and 49.8% beta-glucosidase activity were recovered with glucose concentration reduced to about 15.6 g/L.

It was observed that the *Gelidium* hydrolysate after enzymatic hydrolysis shows some viscosity that is due to presence of residual agar.

### 3.5 *Gelidium* residues derived glucose as promising substrate for P3HB production

**Figure 5:**
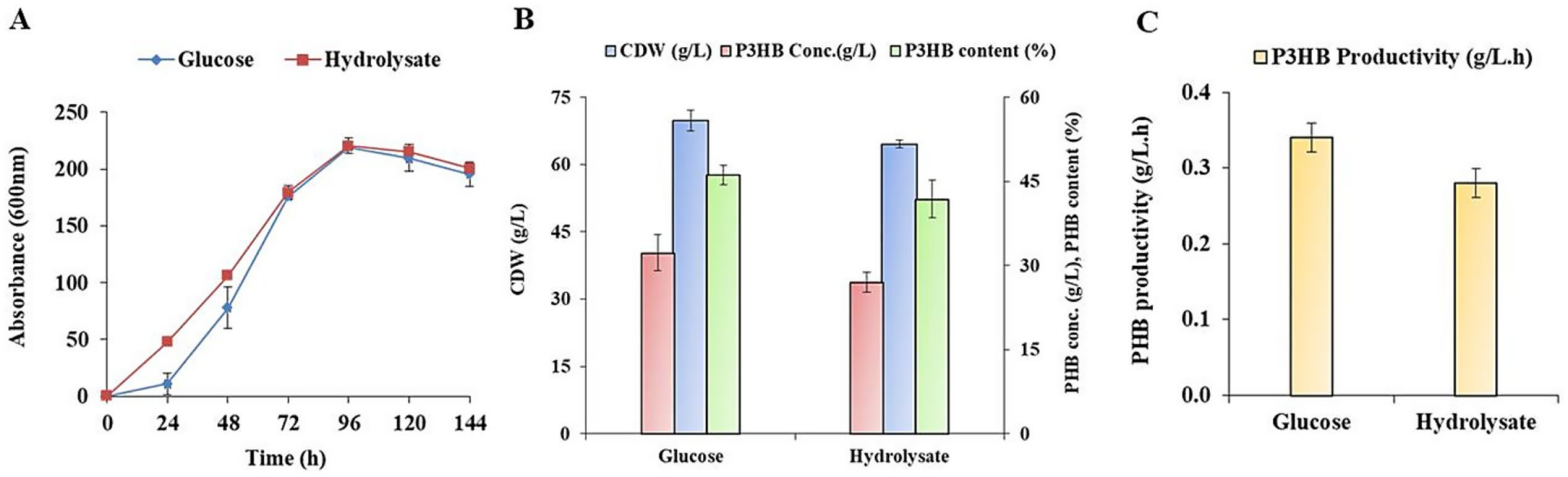
*Halomonas boliviensis* growth and P3HB production profile on *Gelidium* hydrolysate and standard glucose. **(A)** Time course of growth of *H. boliviensis* (absorbance at 600 nm). **(B)** Comparison of CDW (g/L), P3HB concentration (g/L) and P3HB contents (%) of H. boliviensis cultivated on *Gelidium* hydrolysate vs. standard glucose after 96 h cultivation. **(C)** Overall maximum P3HB volumetric productivity achieved with standard glucose and *Gelidium* hydrolysate at 96 h cultivation.

*Gelidium* hydrolysate was evaluated as an alternative carbon source for the growth and P3HB production by *H. boliviensis*, aiming to reduce carbon source costs while promoting the utilization of available C- rich residual raw materials. The *Gelidium* hydrolysate generated by enzymatic hydrolysis (section 2.6) was concentrated using lyophilisation aiming at its preservation. The average glucose content in the powder was 25.1 ± 1.7 g/100 g powder. To use it as a carbon source in feed 1 instead of commercial glucose (Table 1), a concentrated hydrolysate solution containing maximum 230 g/L glucose was obtained. The glucose concentration could not be higher due to viscosity issues that would hinder its use as feed 1. The hydrolysate was also analyzed for hydroxymethylfurfural (HMF), a known inhibitor of bacterial growth frequently released during hydrolysate production and derived from glucose degradation. Its concentration was found to be negligible (0.0084 g/L). To enable a fair comparison of the performance of the *Gelidium* hydrolysate as glucose supplier with that of commercial glucose, a control assay using 230 g/L glucose in feed 1 was carried out. Due to the lower glucose concentration, a bench-scale bioreactor with 5 L working volume was chosen aiming at attaining a higher P3HB production. The results are shown in Figure 5. Similar biomass growth was attained in the control run with CDW of 69.8 g/L versus CDW of 64.6 ± 0.9 g/L with the *Gelidium* hydrolysate after 96 h EFT (Figure 5). The P3HB production was observed to be slightly better in the control with P3HB concentration of 32.3 g/L compared to that of hydrolysate with 27.0 g/L of P3HB corresponding to polymer contents of 46.2 and 41.9%, respectively (Figure 5). The maximum P3HB volumetric productivity achieved at 96 h EFT was 0.34 g/(L·h) for control versus 0.28 g/(L·h) for hydrolysate (Figure 5). The hydrolysate produced from the *Gelidium* residues was thus shown to be a good alternative C-source for the production of P3HB.

## 4 Discussion

In recent years, moderate halophiles have gained importance in P3HB production due their broad substrate range and simpler cultivation conditions. Multiple studies have shown the potential of *H. boliviensis* for P3HB production on simple substrates (Kumar et al., 2012; Torrejon et al., 2025; Miranda et al., 2023; Rivera-Terceros et al., 2015). Our earlier studies on P3HB production by *H. boliviensis* at 2 L scale (Bondar et al., 2022) had achieved a CDW of 49.5 ± 4.9 g/L with maximum P3HB production of 30.4 ± 0.7 g/L, P3HB content of 62.2 ± 7.3% and P3HB volumetric productivity of 0.45 ± 0.03 g/(L·h). To improve overall P3HB volumetric productivity, a change in media composition and feeding strategies were attempted systematically and sequentially, the first of which was focused on the nitrogen source, namely MSG concentration and approach to NH_4_Cl addition.

Glutamate metabolism plays a critical role in nitrogen assimilation by several microbes (Balderrama-Subieta and Quillaguamán, 2013) and *H. boliviensis* has been shown to have restricted growth when glutamine or glutamate is excluded from the growth medium (García-Torreiro et al., 2016). These findings are consistent with previous assays conducted by our group with *H. boliviensis*, where different MSG concentrations in the cultivation medium were evaluated to a maximum of 20 g/L. Increased concentrations of MSG added via feed 2 aimed at promoting cell growth and thus the rate of polymer production. Feed 2 also contained NH_4_Cl. The addition of total NH_4_Cl (6 g/L) was done gradually to avoid cell growth inhibition in the first stages immediately after inoculation. Previous reports by other authors observed that low initial concentration of NH_4_Cl during the growth of *H. boliviensis* led to higher P3HB contents (García-Torreiro et al., 2016; Quillaguamán et al., 2006). Considering this scenario, the controlled feeding of NH_4_Cl along with extra MGS as Feed 2 was carried out during the first 9 h of cultivation. Although this resulted in a 30% increase in P3HB concentration compared to the control run (Figure 1) a significant improvement of cell growth or polymer productivity was not observed (Table 3). This revealed that cell growth rate was not impaired by NH_4_Cl initial concentration nor by the concentration of glutamate in the medium. Nevertheless, Feed 2 was used in the following cultivations because a higher P3HB concentration was attained with this strategy.

The supplementation of a trace element solution to the cultivation medium of *H. boliviensis* has not been carried out before contrarily to the reported for other *Halomonas* species (Leandro et al., 2023; Ren et al., 2018). The TES solution in this study (Table 2) was the one used in the cultivation medium of *H. elongata* (Leandro et al., 2023). Its addition did not improve residual biomass (Xr) at 144 h of cultivation (26.6 ± 3.1 g/L in the control vs. 26.1 ± 2.53 g/L with TES). However, the rate of polymer production showed a significant increase after 72 h cultivation with concentration and content from 24.8 ± 0.6 g/L and 55.4 ± 3.7% in the control run to 37.6 ± 2.4 and 61.6 ± 0.3%, respectively after TES addition. The overall maximum volumetric P3HB productivity increased from 0.34 ± 0.01 g/(L·h) to 0.52 ± 0.03 g/(L·h) after 72 h of cultivation. The addition of trace elements plays thus an important role in production of PHAs by *H. boliviensis* although it has never been considered before.

Finally, the 2-fold increase of the initial phosphate concentration in the medium to 3.0 g/L resulted in a significantly enhanced biomass growth with a Xr = 35.2 ± 0.9 g/L attained after 72 h (20.0 ± 2.5 g/L in the control run) and an improved P3HB production rate resulting in a P3HB concentration of circa 66.0 ± 3.2 g/L thus showing that the rate of biomass growth and in particular of polymer synthesis was limited by the available phosphate in the medium. Some authors report that for maximum polymer production a minimum concentration of phosphorus should be maintained in the medium rather than complete elimination (Castagnoli et al., 2025). Absolute absence of P can cause cell lysis and inhibit P3HB production, because ATP is needed for the conversion of the carbon source to the polymer. An increased overall P3HB productivity of 0.92 ± 0.045 g/(L·h) was obtained by doubling the P concentration in the medium.

Due to their ability to tolerate high salt concentrations in the growth medium (Luo et al., 2022), halophilic bacteria can be grown in less strict sterile conditions and thus processes with these bacteria can save costs on the energy spent with sterilization. Halophiles like *H. campaniensis* LS21 (Yue et al., 2014) and *H. bluephagenesis* TD (Tan et al., 2011) have been cultivated under open unsterile cultivation processes for biopolymer production. The feasibility of carrying out the cultivation of *H. boliviensis* in non-aseptic conditions was assessed using the optimized medium composition and the N-source feeding strategy. The results on Figure 2 show that the results are comparable with the cultivation under sterile conditions. These results are promising because they show the potential of performing cultivations with *H. boliviensis* in non-sterile conditions and attain a polymer productivity of 0.87 ± 0.02 g/(L·h) that is very similar to 0.92 ± 0.045 g/(L·h) in aseptic conditions. This greatly decreases the costs related with sterility matters turning P3HB production with this halophile more competitive compared with non-halophilic microorganisms (Tan et al., 2011).

Besides maintaining sterility, the cost of raw materials- particularly the carbon source-contributes significantly to the overall cost of P3HB production (Xue et al., 2012). The utilization of inexpensive and readily available industrial or agricultural waste residues is expected to reduce the cost of C-source substantially for large-scale fermentation. Utilization of lignocellulosic materials as a substrate for fermentation requires hydrolysis of its carbohydrates, releasing sugars for bacterial metabolism. Before the enzymatic hydrolysis, a pretreatment (hydrothermal or chemical) is needed that alters the structure of cellulosic material and makes it more susceptible to enzymatic degradation (Qi et al., 2012). Pretreatments must be uncomplicated, with no formation of inhibitor compounds and inexpensive to make the overall process economical. However, enzyme cost is usually a major bottleneck in large-scale applications. Therefore, a way to reduce enzyme cost is enzyme recycling (Qi et al., 2012). In this study, enzymatic hydrolysis of pretreated *Gelidium* biomass residue from agar industry was carried out to generate a glucose-rich hydrolysate. The glucose in the hydrolysate was successfully separated from the enzyme using ultrafiltration membrane to recycle the enzyme for the successive enzymatic hydrolysis. The activity of the recovered enzyme decreased with every cycle as can be seen in Figure 4. This could have been due to the loss of stabilizers present in the enzyme cocktail in each recycling step (it is known that sugars are used as stabilizers in enzymatic cocktails) but also with the loss of the protein due to the presence of residual agar in the pre-treated biomass. The enzyme might have been trapped within the agar molecules along with its adsorption on the surface of the biomass. Therefore, to achieve better enzyme recovery, it is essential to optimize the *Gelidium* biomass pretreatment to completely remove the residual agar left in the biomass.

The *Gelidium* hydrolysate examined here as a glucose source for P3HB production by *H. boliviensis* was produced without the recovery of the cellulase cocktail by ultrafiltration. This lyophilized *Gelidium* hydrolysate had 25% (25.1 ± 1.7 g glucose/100 g powder) glucose content. Due to a limited solubility of the powder probably by the presence of agar oligosaccharides and proteins, a maximum concentration of 230 g_glucose_/L solution was obtained. This was used as feed 1 in the fed-batch cultivation instead of the 600 g_glucose_/L in the feed with commercial sugar (Table 1). Because of the dilution caused by a more diluted feed, a bioreactor with a larger volume (5 L working volume) was used. This ultimately resulted in a lower cell density and P3HB titer due to the dilution effect. To be able to compare the results, a control in all similar to the assay with the hydrolysate but using glucose as feed 1 (230 g/L glucose), was carried out. The results in Figure 5 show comparable final P3HB titers at the end of cultivation as the simulated run, namely 27.0 g/L versus 32.2 g/L P3HB, respectively. This suggests that the *Gelidium* hydrolysate can be used as a successful replacement for commercial glucose for P3HB production. To improve *Gelidium* hydrolysate composition, one possible approach could be ultrafiltration to remove the protein fraction (including cellulases) after enzymatic hydrolysis followed by nanofiltration to achieve a 4-5 fold concentration of the hydrolysate.

In conclusion, the growth and P3HB production of the moderately halophilic bacterium *H. boliviensis* were significantly improved through modifications of the culture medium composition and feeding strategy. An overall volumetric productivity of 0.92 ± 0.045 g/(L·h) was achieved along with maximum P3HB concentration of 70.9 ± 0.3 g/L which are considerably higher than previously reported with *H. boliviensis*. To reduce the overall production cost of P3HB two strategies were successfully assessed namely the cultivation in non-aseptic conditions and the use of glucose-rich hydrolysates produced by enzymatic hydrolysis from seaweed industrial by-products. To decrease costs associated with the enzyme, the recycling of the CellicCtec3 HS cellulase cocktail using ultrafiltration is feasible, but the enzyme activity decreases circa 60% after 4 cycles. Cultivations with *H. boliviensis* for P3HB production using an improved medium composition, non-sterile conditions and non-food residual biomasses as sugar providers are promising and competitive systems to produce the poly-3-hydroxybutyrate microbial polyester.

## Data Availability

The raw data supporting the conclusions of this article will be made available by the authors, without undue reservation.

## Supplementary material

SUPPLEMENTARY FIGURE S1 *H. boliviensis* growth and P3HB production profile in fed-batch cultivation in medium BM2 with controlled feeding of nitrogen source (NH_4_Cl and MSG). **(A)** Cell dry weight (CDW), residual cell concentration (Xr), P3HB concentration and P3HB content obtained during the cultivation. **(B)** Glucose and gluconic acid concentration.

SUPPLEMENTARY FIGURE S2 *H. boliviensis* growth and P3HB production profile in fed-batch cultivation in BM3 medium with Trace Elements Solution **(A)** Cell dry weight (CDW), residual cell concentration (Xr), P3HB concentration and P3HB content achieved during the cultivation. **(B)** Glucose and gluconic acid concentration.
