# Supplementary figures and images for: Towards economic and sustainable production of poly-3-hydroxybutyrate by Halomonas boliviensis using as feedstock industrial residues of seaweed Gelidium corneum
Source: Front Microbiol. 2026 Jun 25;17:1872452. doi: 10.3389/fmicb.2026.1872452 (PMC13346058; doi:10.3389/fmicb.2026.1872452)

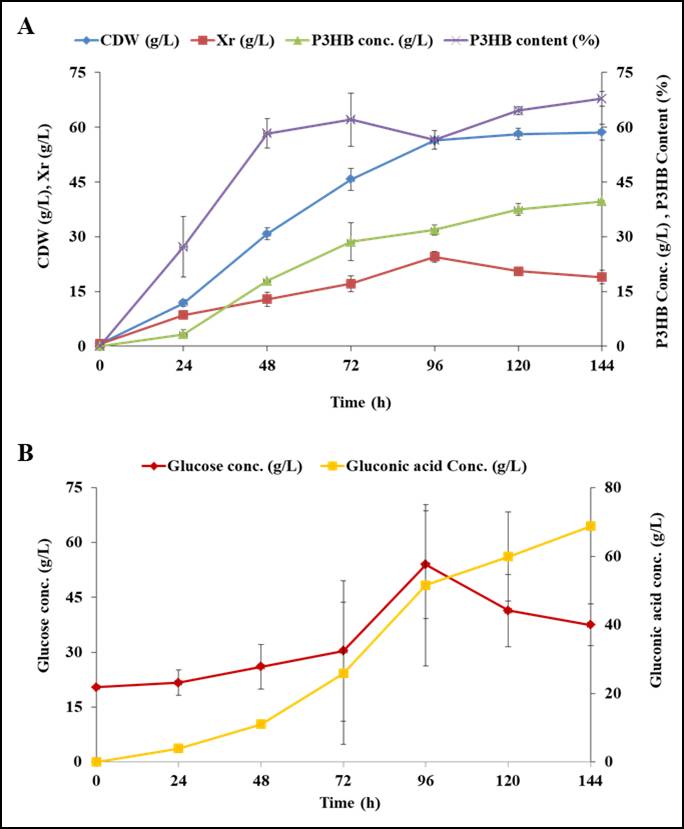

Supplement: SUPPLEMENTARY FIGURE S1 — H. boliviensis growth and P3HB production profile in fed-batch cultivation in medium BM2 with controlled feeding of nitrogen source (NH4Cl and MSG). (A) Cell dry weight (CDW), residual cell concentration (Xr), P3HB concentration and P3HB content obtained during the cultivation. (B) Glucose and gluconic acid concentration. [file Image_1.JPEG]

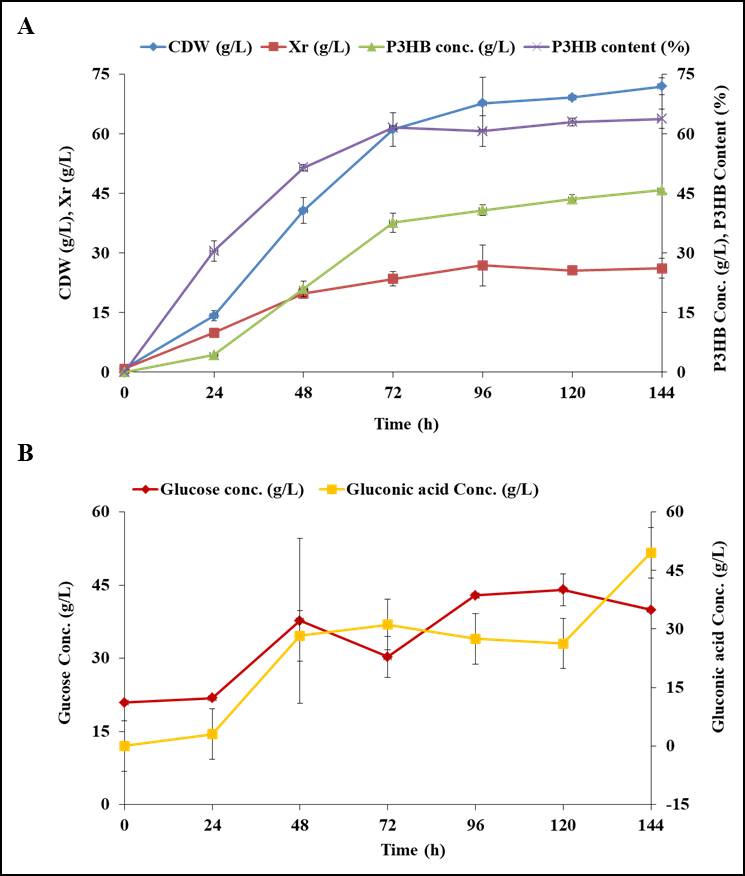

Supplement: SUPPLEMENTARY FIGURE S2 — H. boliviensis growth and P3HB production profile in fed-batch cultivation in BM3 medium with Trace Elements Solution (A) Cell dry weight (CDW), residual cell concentration (Xr), P3HB concentration and P3HB content achieved during the cultivation. (B) Glucose and gluconic acid concentration. [file Image_2.JPEG]
